# Subject-Specific Finite Element Modelling of Le Fort I Osteotomy Fixation: Biomechanical Insights Comparing Patient-Specific Implants and Conventional Miniplates

**DOI:** 10.3390/jcm15207994

**Published:** 2026-10-15

**Authors:** Olina Rios, Sarah Latreche, Yannick Tillier, Charles Savoldelli

**Affiliations:** 1 Department of Oral and Maxillo-Facial Surgery, Head and Neck Institute, University Hospital Center of Nice, 31 avenue de Valombrose, 06100 Nice, France; latreche.s@chu-nice.fr (S.L.); savoldelli.c@chu-nice.fr (C.S.); 2 Nice Côte d’Azur Clinical Research Unit (UR2CA), 28 avenue Valombrose, 06107 Nice Cedex 2, France; 3 Department of Computational Mechanics Physics CEMEF, MINES ParisTech, PSL Research University, Centre de Mise en Forme des Matériaux (CEMEF), 06904 Sophia Antipolis, France; yannick.tillier@minesparis.psl.eu

**Keywords:** 3D printed plates, biomechanics, finite element analysis, orthognathic surgery, patient-specific implants

## Abstract

**Background**: Stable fixation is essential to prevent relapse after Le Fort I osteotomy, particularly during the early postoperative period when the fixation system bears the full masticatory load. Patient-specific implants (PSI) have been increasingly used, but their biomechanical behavior under postoperative surgical conditions remains insufficiently investigated. The aim of this study was to evaluate PSI fixation using subject-specific finite element modelling based on real surgical plans. **Methods**: Two patient-specific models were developed from cone-beam computed tomography (CBCT) data of patients undergoing bimaxillary orthognathic surgery. For each case, two fixation strategies were simulated: a multiarm patient-specific plate and conventional L-shaped miniplates. Von Mises stress distribution and relative displacement were analyzed for fixation devices and bone segments. **Results**: PSI fixation reduced stress levels in plates and screws while maintaining comparable global stability. However, its effect on bone stress differed between the two models. In Patient 2, whose surgical plan included greater maxillary advancement and posterior impaction, PSI fixation was associated with higher bone stress and a micrometric increase in displacement. **Conclusions**: These findings highlight the influence of surgical movement on fixation biomechanics and support the integration of biomechanical personalization alongside anatomical planning in orthognathic surgery.

## 1. Introduction

Le Fort I osteotomy is a standard procedure for the correction of dentofacial deformities, allowing controlled repositioning of the maxilla. The Le Fort I osteotomy takes its name from the horizontal maxillary fracture pattern described by René Le Fort in 1901, although horizontal maxillary osteotomy had already been described by von Langenbeck in 1859 for temporary maxillary mobilization. Its application to the correction of dentofacial deformities developed later, with Wassmund performing total maxillary osteotomy for correction of midfacial malposition in 1927 and subsequent refinements progressively leading to the modern Le Fort I procedure [1]. The contemporary procedure consists of a horizontal osteotomy above the dental apices, extending through the lateral and medial maxillary walls to the pterygomaxillary junction, followed by down-fracture and three-dimensional repositioning of the tooth-bearing maxillary segment according to the surgical plan. The repositioned maxilla is then stabilized by osteosynthesis, making fixation mechanics particularly relevant during the early postoperative period before bone healing occurs, when the fixation system bears full masticatory load.

Many studies have assessed the strength and stability of different fixation strategies for Le Fort I osteotomy [2,3,4]. Currently, the gold standard is fixation with four conventional L-shaped miniplates, as this type of fixation has been shown to be the most stable with minimal stress on the plates [5,6,7]. The development of computer-assisted surgical planning and computer-aided design/computer-aided manufacturing (CAD-CAM) technologies has enabled the fabrication of patient-specific implants (PSIs), introducing new fixation strategies for maxillary and mandibular osteotomies. Early approaches included “prebent” plates [8], still relying on four separate plates, and later the emergence of a multiarm titanium single plate (Materialise, Malakoff, France) manufactured by selective laser melting and now widely used by maxillo-facial surgeons throughout Europe [9,10,11]. This PSI is numerically created prior to surgery via a numerical reverse process: first, the screws are placed in areas of optimal bone thickness above the tooth roots; then, they are connected by bridges reproducing traditional plate locations and finally merged into a unified multiarm plate. Unlike conventional miniplates -which are ductile and require intraoperative contouring- PSIs are not intended to be bent by hand. Their rigidity and patient-specific shape enable them to guide the maxilla into its planned preoperative position. In contrast, repeated bending of conventional plates may introduce residual stresses at their bending area, as this particular zone carries the most constraints, increasing the risk of fatigue failure [12,13].

The incidence of relapse after orthognathic surgery depends on both fixation configuration and the magnitude of maxillary advancement [14,15,16]. By enveloping the maxilla in a cage-like structure, the multiarm PSI may provide improved rigidity and support for masticatory forces, potentially benefiting large advancements or segmental Le Fort I osteotomies where maintaining transverse dimension is challenging [17]. Conversely, excessive rigidity in one-piece osteotomies may limit natural micromovements essential for bone healing. Furthermore, forcefully linking two embryologic maxillary halves might lead to stress accumulation. Previous studies have demonstrated that patient-specific maxillary single plates are an efficient alternative to traditional plates in terms of stability [18,19] and precision [20,21] in maxillary repositioning. However, to our knowledge, no study has investigated their mechanical behavior and consequences on bone segments.

Finite element analysis (FEA) allows the patient-specific plate to be evaluated and compared to the gold standard fixation configuration in the same patient. With the advent of FEA, surgeons have gained tools to virtually perform osteotomies and bone relocations, test the optimal positions, strains and stresses of fixation materials [22], understand the mechanical consequences of surgery [23,24,25] and improve the type and shape of the material used [13,26,27]. Prior finite element (FE) studies have explored fixation strategies for Le Fort I osteotomy [7,26,28,29,30,31,32], but none have examined the multiarm PSI. Moreover, no previous work has integrated the actual treatment plan (planned bone relocation) into biomechanical simulation. Implementing the real treatment plan allows a personalized assessment of the mechanical consequences of surgery, complementing anatomical personalization with biomechanical personalization.

Accordingly, this study aimed to evaluate the biomechanical performance of patient-specific versus conventional fixation for Le Fort I osteotomy using subject-specific finite element models derived from real surgical plans. Two distinct clinical scenarios—a minor advancement without impaction and a moderate advancement with posterior impaction—were analyzed to determine how surgical movements influence implant stress, bone stress, and maxillary micromotion. By combining surgical planning with subject-specific computational modeling, this work provides clinically relevant insights to optimize PSI design and guide fixation strategy selection in orthognathic surgery.

## 2. Materials and Methods

The dataset used in this study consisted of cone-beam computed tomography (CBCT) scans (field of view: 22 × 16 cm; scan time: 40 s; voxel size: 0.4 mm) acquired using an I-CAT 3D Imaging System (Imaging Sciences International, Inc., Hatfield, PA, USA) from two adult patients who underwent bimaxillary orthognathic surgery at the University Hospital Center of Nice. These imaging datasets were retrospectively selected from the institutional clinical imaging archive and were fully anonymized prior to analysis. The two cases were selected to represent two common dentofacial malocclusion patterns, with one patient presenting a Class II malocclusion and the other a Class III malocclusion. Both underwent bimaxillary orthognathic surgery without associated genioplasty. Both surgical plans included clockwise rotation of the occlusal plane, while patient n°2 also underwent posterior maxillary impaction. Patient characteristics are summarized in Table 1.

**Figure A1:**
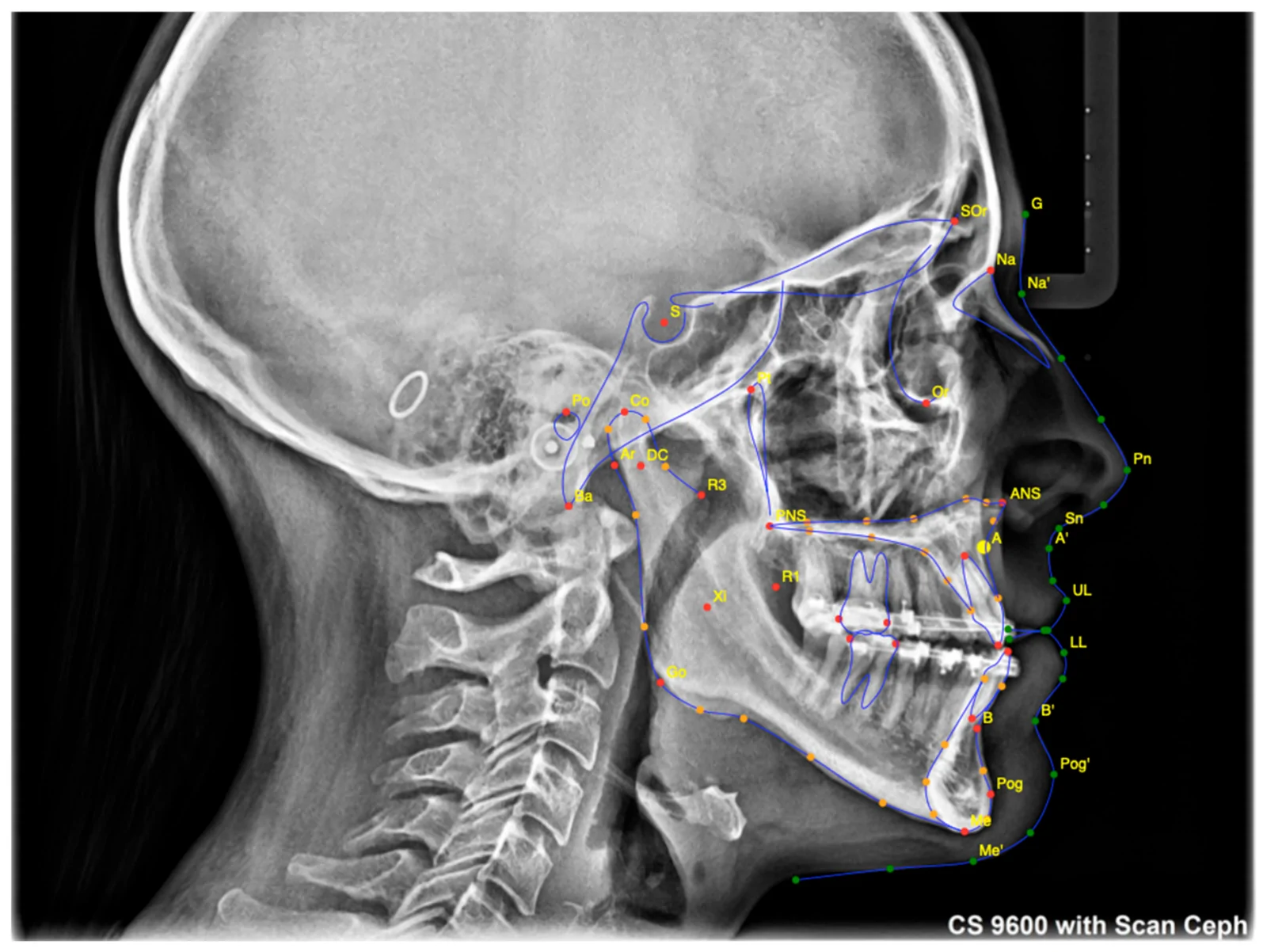
Preoperative cephalometry of patient n°2, analyzed with Webceph (https://webceph.com).

The corresponding CBCT data were exported in Digital Imaging and Communications in Medicine (DICOM) format and imported into Mimics Medical (version 25.0, Materialise, Leuven, Belgium) for segmentation and three-dimensional cranio-maxillofacial reconstruction. All segmentations were performed by the same clinical engineer using voxel-based segmentation to differentiate cortical and cancellous bone structures. For each patient, the Le Fort I osteotomy was designed on both cortical and trabecular bone according to the actual surgical treatment plan using ProPlan CMF software (version 3.0, Materialise, Leuven, Belgium). Geometry preparation for finite element analysis was performed using Meshmixer (Autodesk Meshmixer (RRID:SCR_015736), version 3.5 Autodesk Inc., http://www.meshmixer.com/, date of first access 4 January 2026). Additional methodological details, including planned surgical movements (Table A1) and cephalometric landmarks (Figure A1) are provided in the Appendix A. Surgical planning was based on clinical, occlusal, and cephalometric assessment, with cephalometric evaluation primarily based on Delaire analysis and complemented by modified Steiner parameters.

### 2.1. Plates and Screws

Two fixation systems were modeled: (1) patient-specific maxillary multiarm plates, and (2) conventional 4-hole L-shaped miniplates. The characteristics of the plates are shown in Table 2. All implants and screws were digitally reconstructed in 3-Matic Medical (version 20.0, Materialise, Leuven, Belgium) by the same biomedical engineer ensuring consistency in modeling and comparable plate-to-bone contact conditions between the two configurations. Conventional plates were selected from a digital library of commonly used 4-hole L-shaped designs and numerically adapted to the postoperative bone surface using anatomical landmarks and surface-fitting tools. Although conventional plates are manually contoured intraoperatively in clinical practice, this step was not simulated here, as residual stress from manual bending is not yet quantifiable in finite element studies. Therefore, this modeling choice provides a neutral basis for comparison and may in fact favor the conventional configuration regarding mechanical performance since residual stress cannot be implemented.

**Figure 1:**
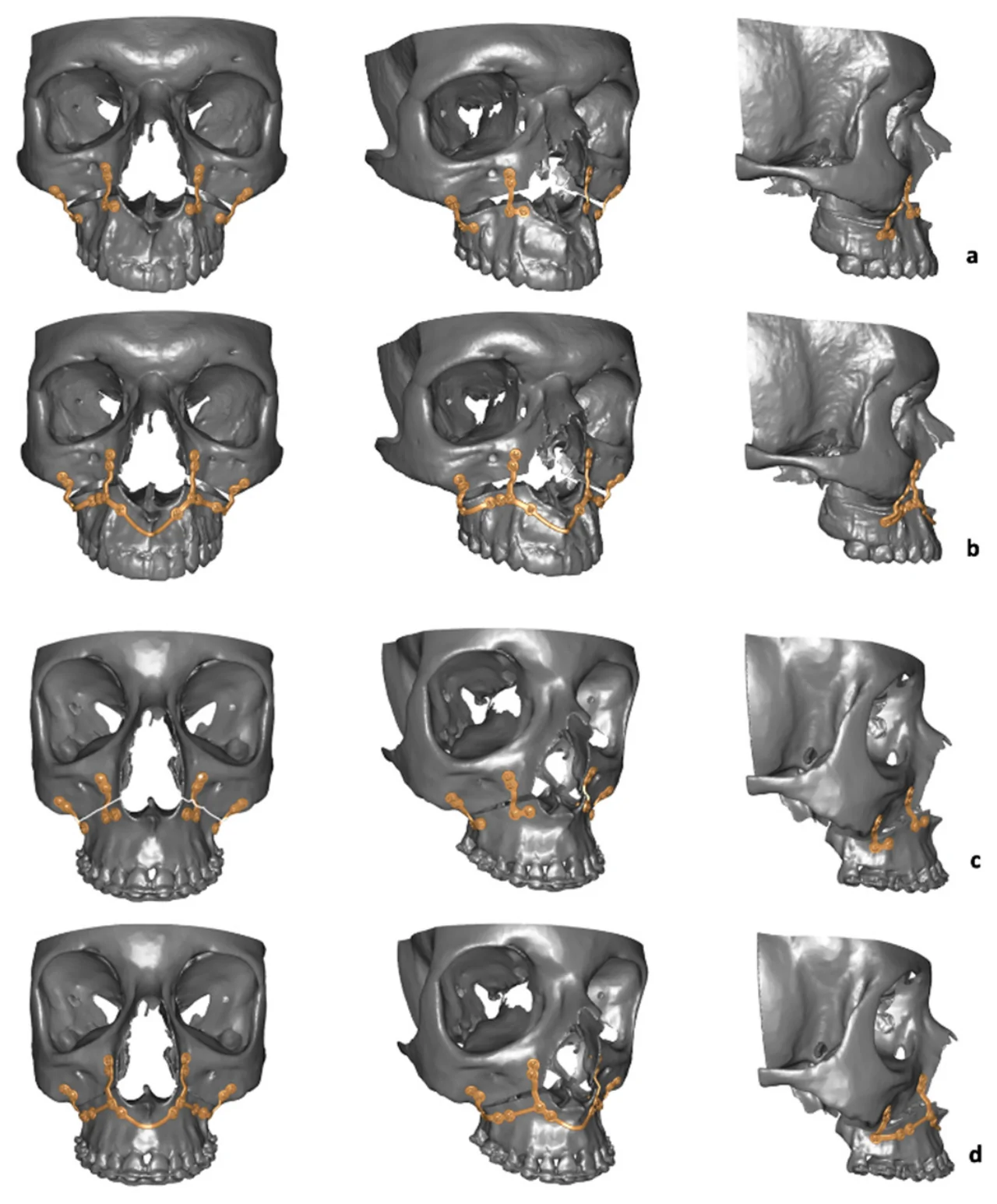
Three-dimensional reconstructions of maxillary fixation configurations in both patients. (**a**) conventional L-shaped miniplate fixation in patient n°1, (**b**) patient-specific multiarm plate fixation in patient n°1, (**c**) conventional L-shaped miniplate fixation in patient n°2, (**d**) patient-specific multiarm plate fixation in patient n°2.

Each patient was virtually operated on with the two types of fixation systems, meaning that their respective three-dimensional skulls were similar except for the fixation method. Conventional miniplates were traditionally placed symmetrically on both sides near the zygomaticomaxillary buttress and the apertura piriformis (Figure 1a,c). The patient-specific plates were individualized according to the patient’s anatomy and bone thickness (Figure 1b,d). Screws were 2.0 mm × 5 mm in both configurations, corresponding to clinical standards in our department.

### 2.2. Establishment of the 3D Finite Element Model (FEM)

**Figure 2:**
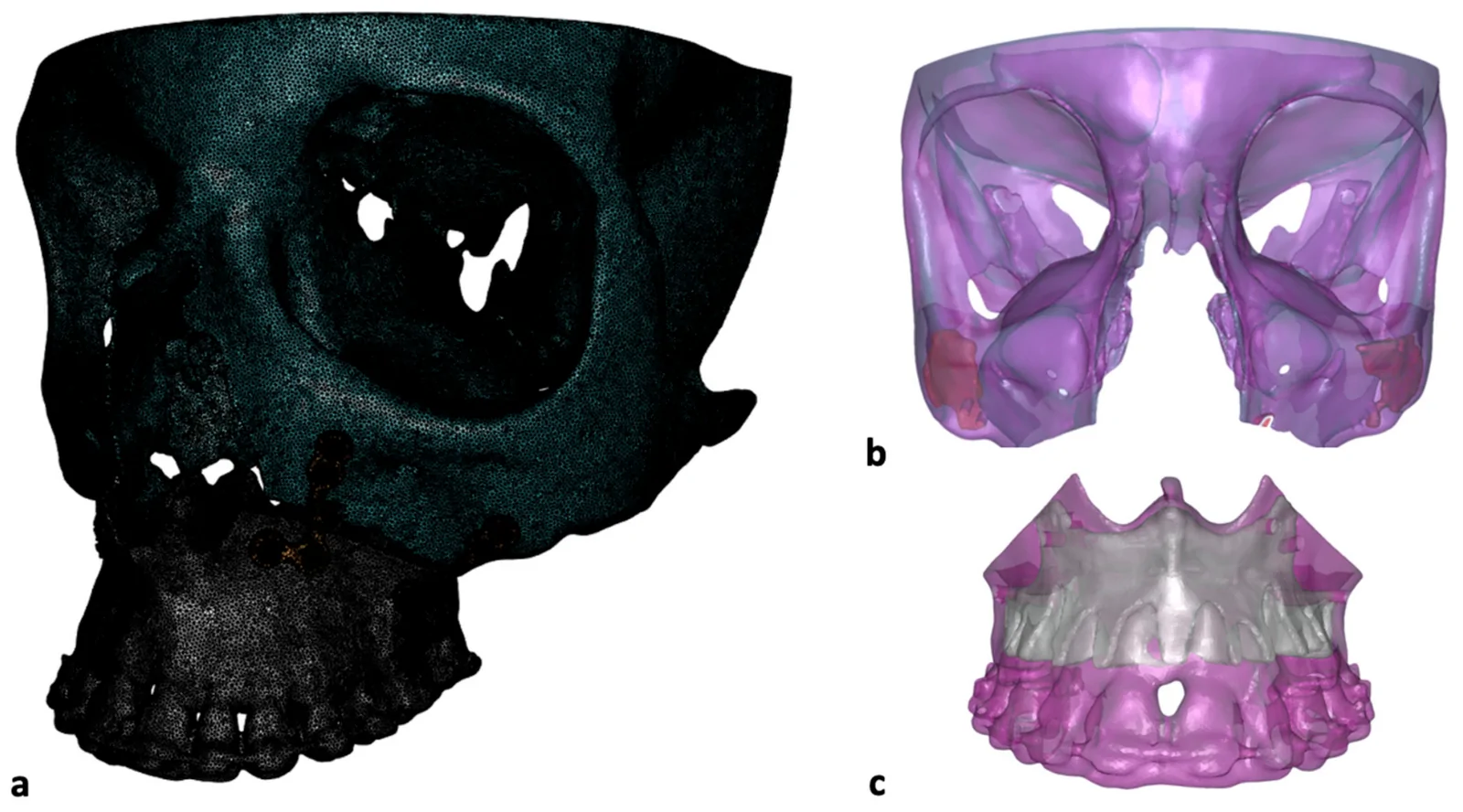
3D modelled objects. (**a**) 3D model of patient n°2 with a mesh size of 0.5 mm, (**b**) 3D model of the midface and the maxilla (**c**) showing cancellous bone parts (in red and gray) by transparency.

All the modeled objects (bone and implants) were exported as standard tessellation language (STL) files into the FORGE NxT 4.0 finite element software (Transvalor, Biot, France), which has already been used for biomechanical modeling [33,34,35,36]. Each 3D model was made of three distinct objects: the maxilla (with cortical and trabecular bone), the midface (with cortical and trabecular bone) and a complex plate + screws. A mesh convergence test was carried out prior to the study to find the ideal mesh size allowing convergence of the calculations and the best evaluation of the maximum von Mises stress values and relative displacements between bone segments. A 0.5 mm surface mesh was established, and tetrahedral elements (P1+/P1) were used to generate the corresponding volume mesh (Figure 2). Contact types were defined in accordance with previous studies [27]. A multimaterial approach was used to model cortical and cancellous bones. Unlike the multiobject approach also available in FORGE, this choice allows only one mesh to be considered for the bone tissues while assigning different constitutive equations to the elements depending on the type of bone considered (cortical or trabecular). This simplifies the management of contact between the two types of bone and drastically reduces computation times. On the other hand, it implies perfect contact between the two (binding—contact relationship), with no possibility of relative movement, which is a priori the case. The plate-screw-bone interface was also considered a fully bonded condition (sticking contact) to allow for the continuity of stress transfer.

A second preliminary test was performed prior to the study to select the contact condition between the bone segments (midface and maxilla). Friction contact with a friction coefficient of 0.5, as used in previous FE studies [7,13,26], was compared with sliding contact during the mesh/contact convergence assessment. Under the tested conditions, sliding contact provided satisfactory force-displacement and von Mises stress convergence while facilitating numerical convergence and reducing computation time. It was therefore selected for the final simulations. In addition, sliding contact permits relative tangential micromotion between the repositioned bone segments after osteotomy, consistent with the mechanical conditions intended to be represented in the immediate postoperative model. All the calculation results were postprocessed and analyzed directly within the FORGE interface. The final numbers of elements and nodes are shown in Table 3.

### 2.3. Material Parameters

All titanium plates and screws were modeled in Ti alloy (unalloyed “pure” Ti40 for conventional plates and Ti6Al4V for PSIs). The material parameters used in the finite element software are shown in Table 4 and were obtained from both the literature and material suppliers. All the materials were assumed to be homogeneous, linearly elastic and isotropic.

### 2.4. Boundary Conditions and Loads

Choi et al. [37] reported the evolution of occlusal forces after Le Fort I osteotomy, with average bite forces of 97.6 N at one month, 206.9 N at three months, and 257 N at six months post-operatively. In light of these data and considering that our finite element model simulates the immediate postoperative period prior to bone healing, we selected a chewing load of 100 N to represent the functional stress experienced by the fixation system during this early healing phase. The forces measured by Choi et al. have been used in several FE studies evaluating Le Fort I osteotomy fixation methods [26,32]. Importantly, the early postoperative phase represents a critical window during which the entire mechanical responsibility for skeletal stability lies on the fixation system (plates and screws). The findings of Sugiura et al. [38] further support this choice, demonstrating that peak stress levels on miniplates occur between 2 and 4 weeks after surgery.

In addition, previous FEM studies have shown that the mechanical behavior of fixation materials varies significantly depending on the loading direction. For example, Ataç et al. [28,29] demonstrated that oblique loading induces greater von Mises stress on miniplates than purely vertical forces do. Consequently, our model uses a combined loading: a compressive force applied at a 45° inclination to the long axis of the premolars and molars, which is directed from palatal to buccal. This loading protocol, adopted in further biomechanical simulations of Le Fort I osteotomy [7,13], better reflects the multidirectional nature of real masticatory forces. The load was applied bilaterally to the molars and premolars, which are the teeth most involved in occlusal function and are located close to the posterior fixation points.

**Figure 3:**
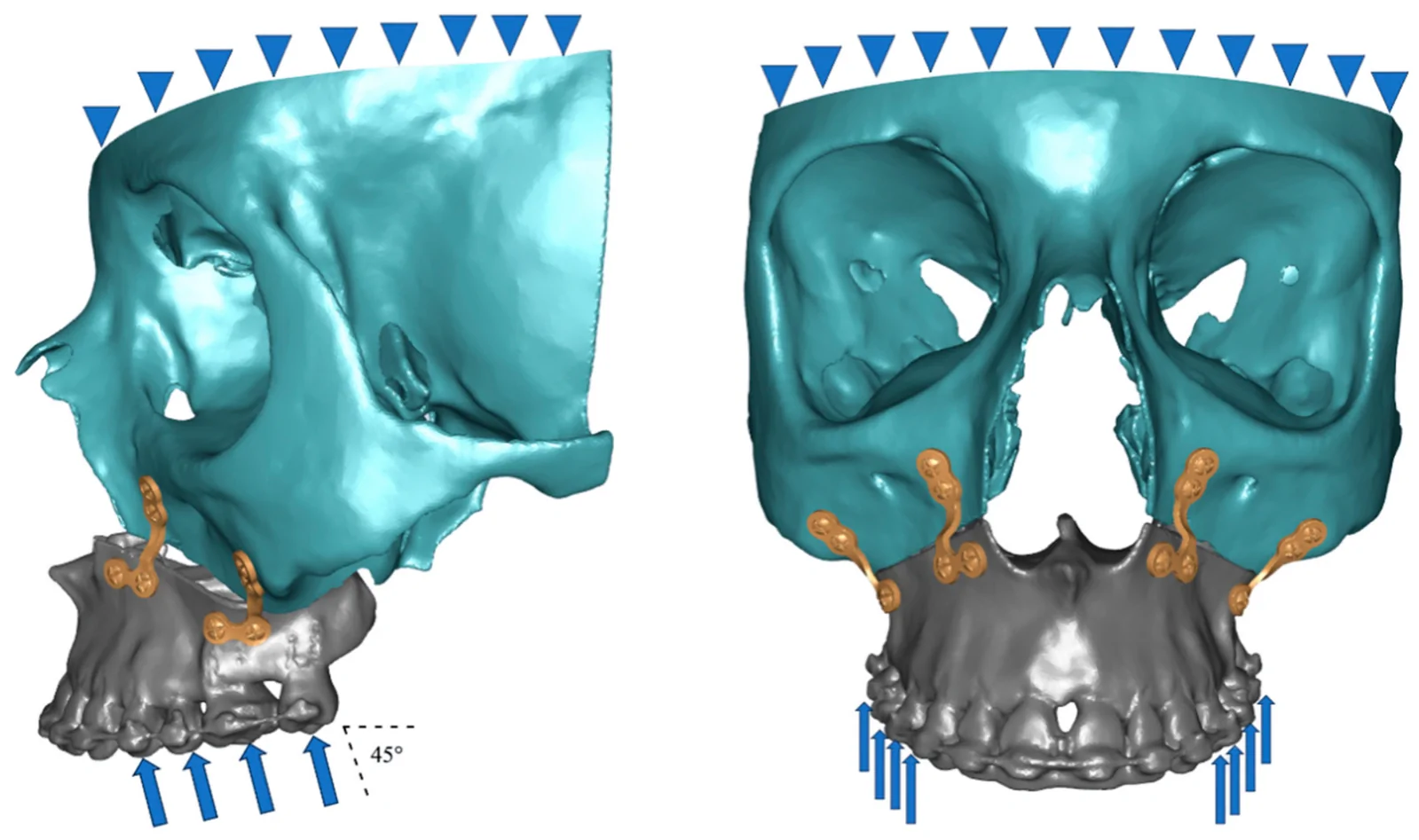
Boundary (triangles) and loading (arrows) conditions applied to the model.

The superior cutting plane of the skull was constrained in all three axes (x, y, z) to ensure model stability. These conditions aim to recreate a clinically relevant loading scenario reflecting the patient’s function during the first postoperative weeks, when the fixation system alone must maintain skeletal stability. The boundary and loading conditions are presented in Figure 3.

### 2.5. Data Analysis

The relative displacement and maximum von Mises stress on the plates, screws and bone segments were assessed for each patient in each fixation configuration.

## 3. Results

### 3.1. Relative Displacement of Bone Segments

**Figure 4:**
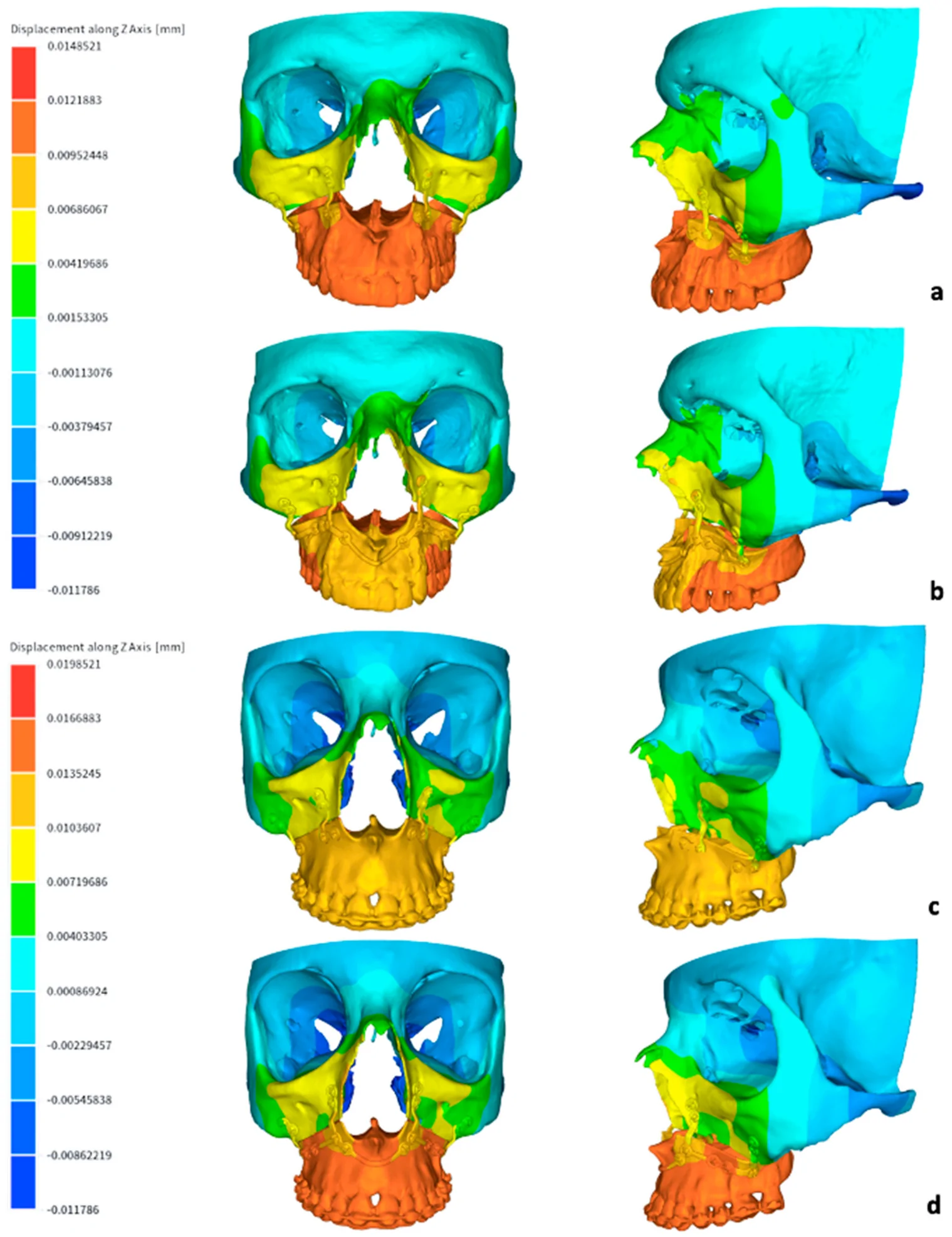
Total relative displacement of the craniofacial models under loading conditions. (**a**) conventional fixation in patient n°1, (**b**) patient-specific fixation in patient n°1, (**c**) conventional fixation in patient n°2, (**d**) patient-specific fixation in patient n°2.

**Figure 5:**
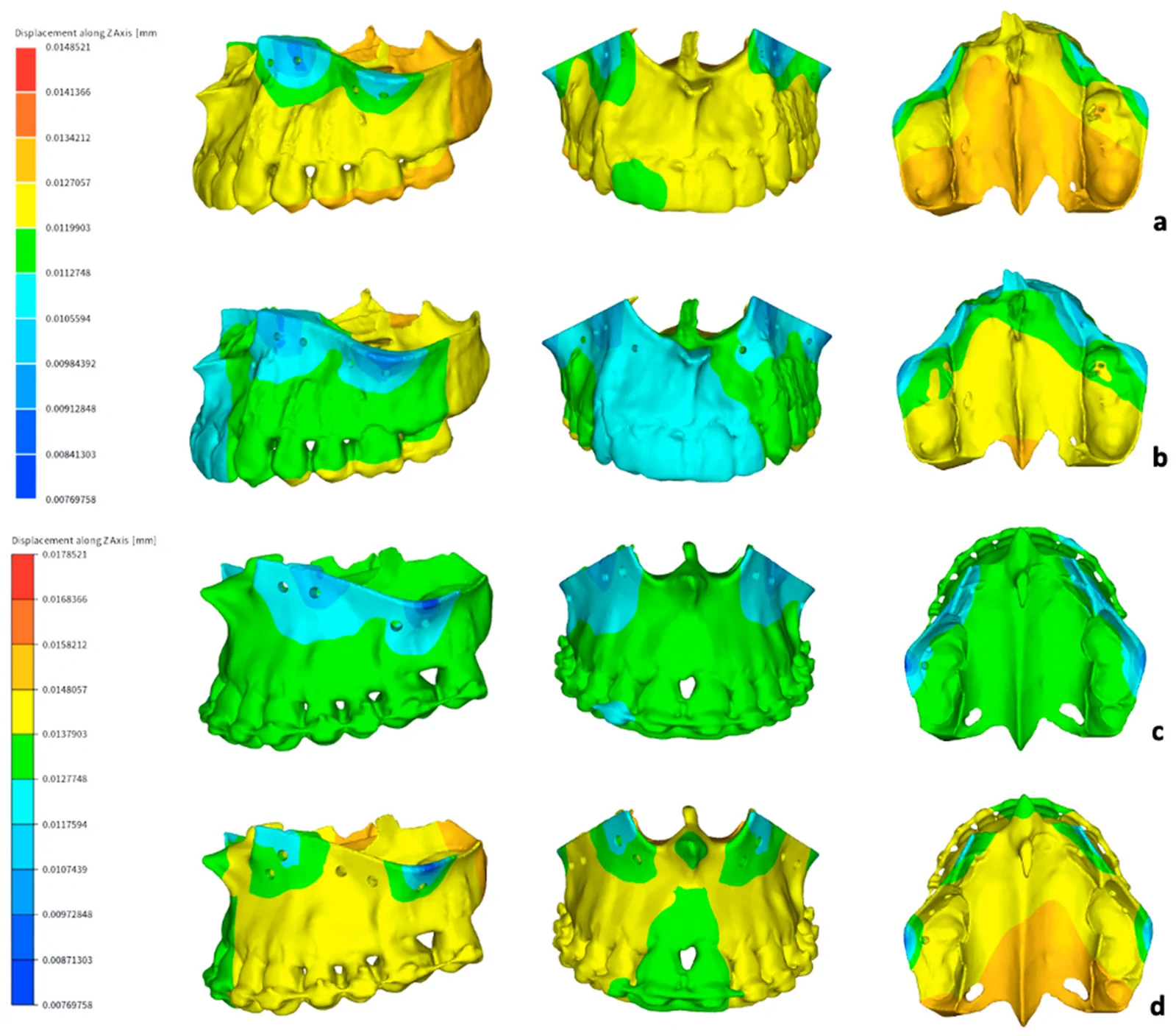
Total relative displacement of the maxilla segment under loading conditions (**a**) conventional fixation in patient n°1, (**b**) patient-specific fixation in patient n°1, (**c**) conventional fixation in patient n°2, (**d**) patient-specific fixation in patient n°2.

Total relative displacement of each model is presented in Figure 4. In patient n°1, PSI fixation slightly improved maxillary stability compared with conventional plates. In patient n°2, PSI generated higher displacement, but the difference remained extremely small (<0.003 mm). For both patients, displacement of the maxilla was mainly concentrated in its posterior region (Figure 5). For all the simulations, the fixation zones exhibited the least displacement. As shown in Table 5, greater maxillary advancement was associated with higher displacement values.

### 3.2. Von Mises Stress Distribution on Plates and Screws

**Figure 6:**
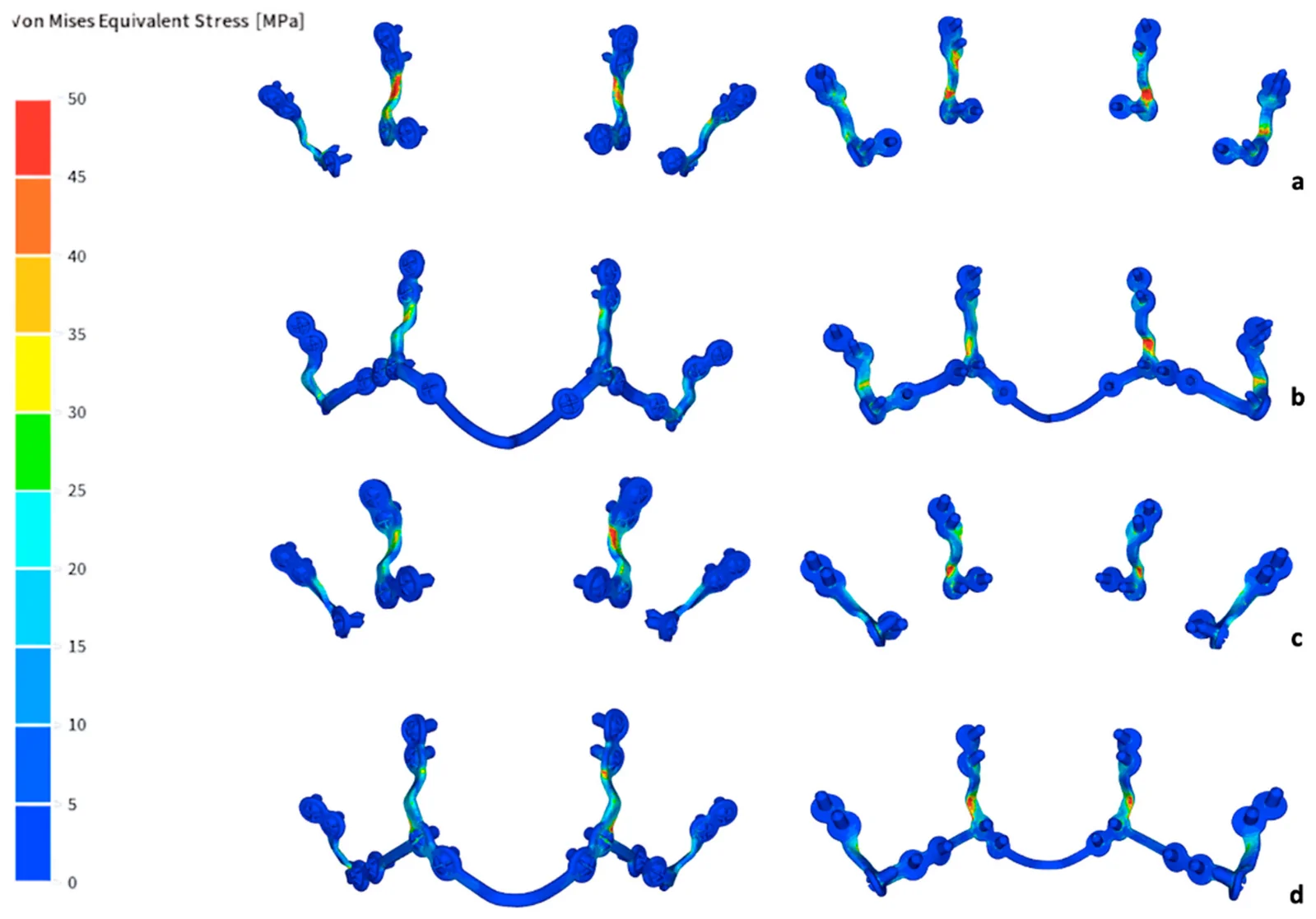
Von Mises stress distribution on the plates and screws for both fixation strategies. (**a**) conventional miniplates in patient n°1, (**b**) patient-specific plate in patient n°1, (**c**) conventional miniplates in patient n°2, (**d**) patient-specific plate in patient n°2.

**Figure 7:**
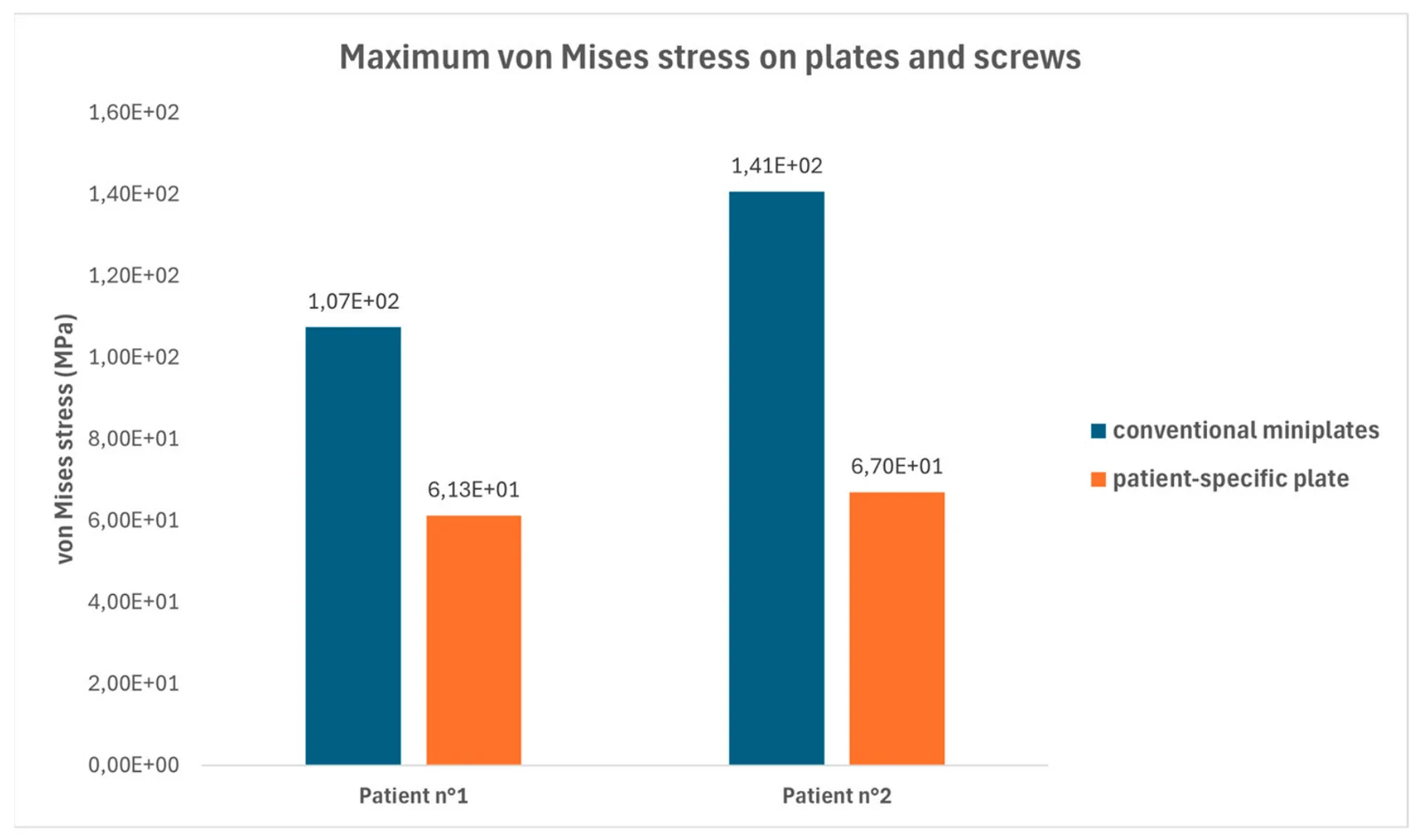
Maximum von Mises stress, in MPa, on the plates and screws.

The von Mises stress distributions on the plates and screws are shown in Figure 6, with numerical values provided in Table 6 and Figure 7. For patient n°1, the maximum von Mises stress reached 107.4 MPa with conventional fixation versus 61.3 MPa with PSI. In patient n°2, the values were 140.7 MPa and 67 MPa, respectively. In all models, stress concentrated in the bent or curved regions facing the osteotomy line. Patient-specific plates exhibited a more uniform stress distribution and lower peak von Mises stress. For both patients and configurations, peak stress values remained below the reported yield strength of the corresponding titanium materials (Table 2). However, as the present analysis was based on static loading, no inference regarding fatigue resistance or cyclic failure can be made.

### 3.3. Von Mises Stress Distribution on Bone Segments

**Figure 8:**
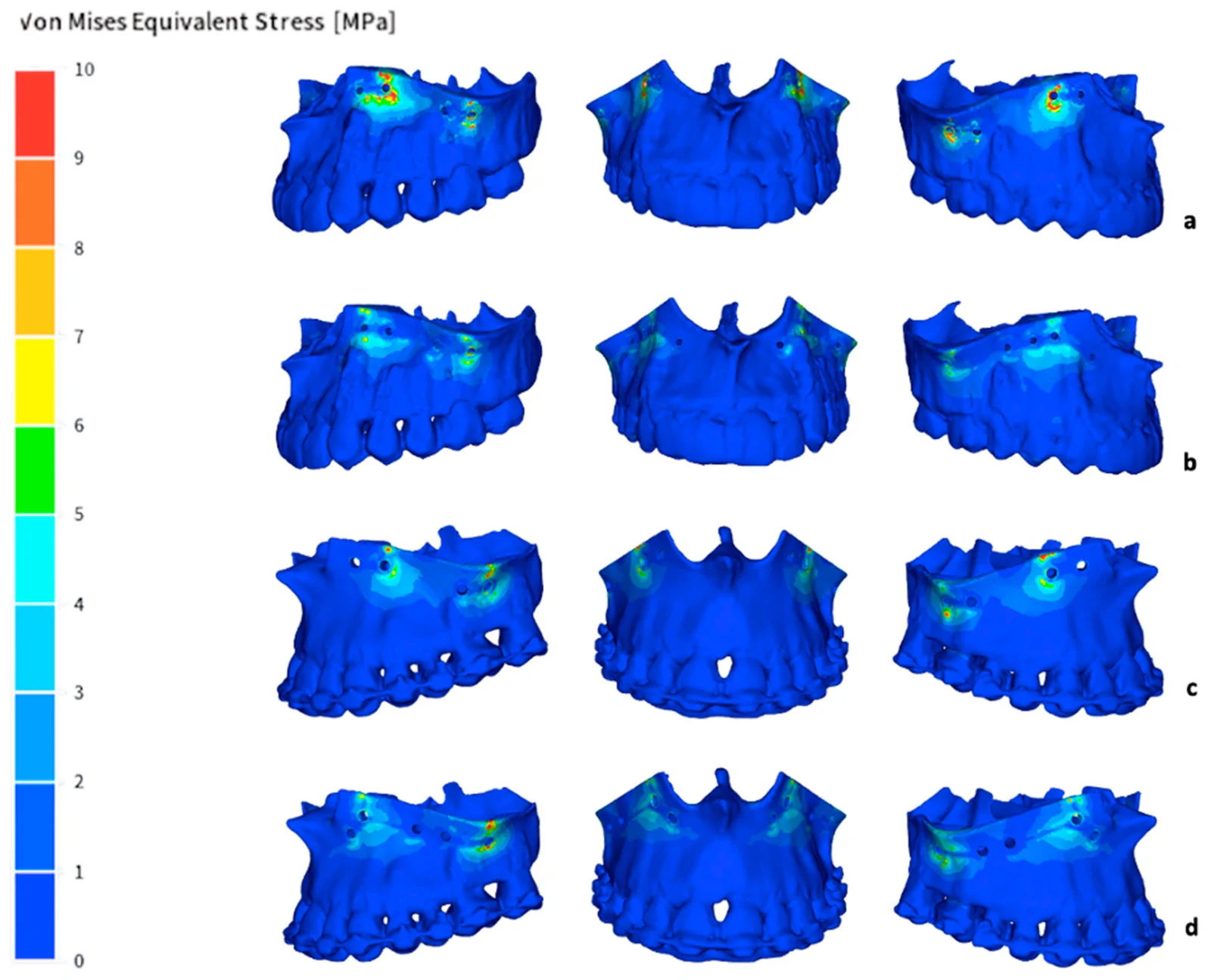
Comparison of stress distribution in the maxilla between fixation configurations. (**a**) conventional fixation in patient n°1, (**b**) patient-specific fixation in patient n°1, (**c**) conventional fixation in patient n°2, (**d**) patient-specific fixation in patient n°2.

Bone stress decreased markedly with PSI fixation in patient n°1 (−54% in the maxilla and −83% in the midface; Table 7). In patient n°2, PSI increased bone stress, although differences remained limited (<15 MPa). In the conventional configuration, stress was mainly concentrated around screw holes, potentially increasing the risk of microfractures and screw loosening. With the PSI fixation, stress spread across broader regions of the nasomaxillary and zygomaticomaxillary buttresses (Figure 8). For both patients, midface stress exceeded maxillary stress, likely reflecting the damping effect of cancellous bone in the maxilla.

## 4. Discussion

The aim of this study was to evaluate the biomechanical consequences of patient-specific fixation for Le Fort I osteotomy by comparing the von Mises stress levels and maxillary displacement with those of the current gold standard, conventional L-shaped miniplates. Using subject-specific finite element models derived from real surgical plans, we found that PSIs substantially reduced peak implant stress while maintaining clinically acceptable maxillary stability. However, reduced stress within the fixation system did not systematically translate into reduced stress within the surrounding bone. While PSI fixation reduced peak implant stress in both models, bone stress decreased markedly in Patient 1 but increased in Patient 2. This dissociation suggests that implant-level and bone-level mechanical responses should both be considered when evaluating fixation strategies.

**Figure A2:**
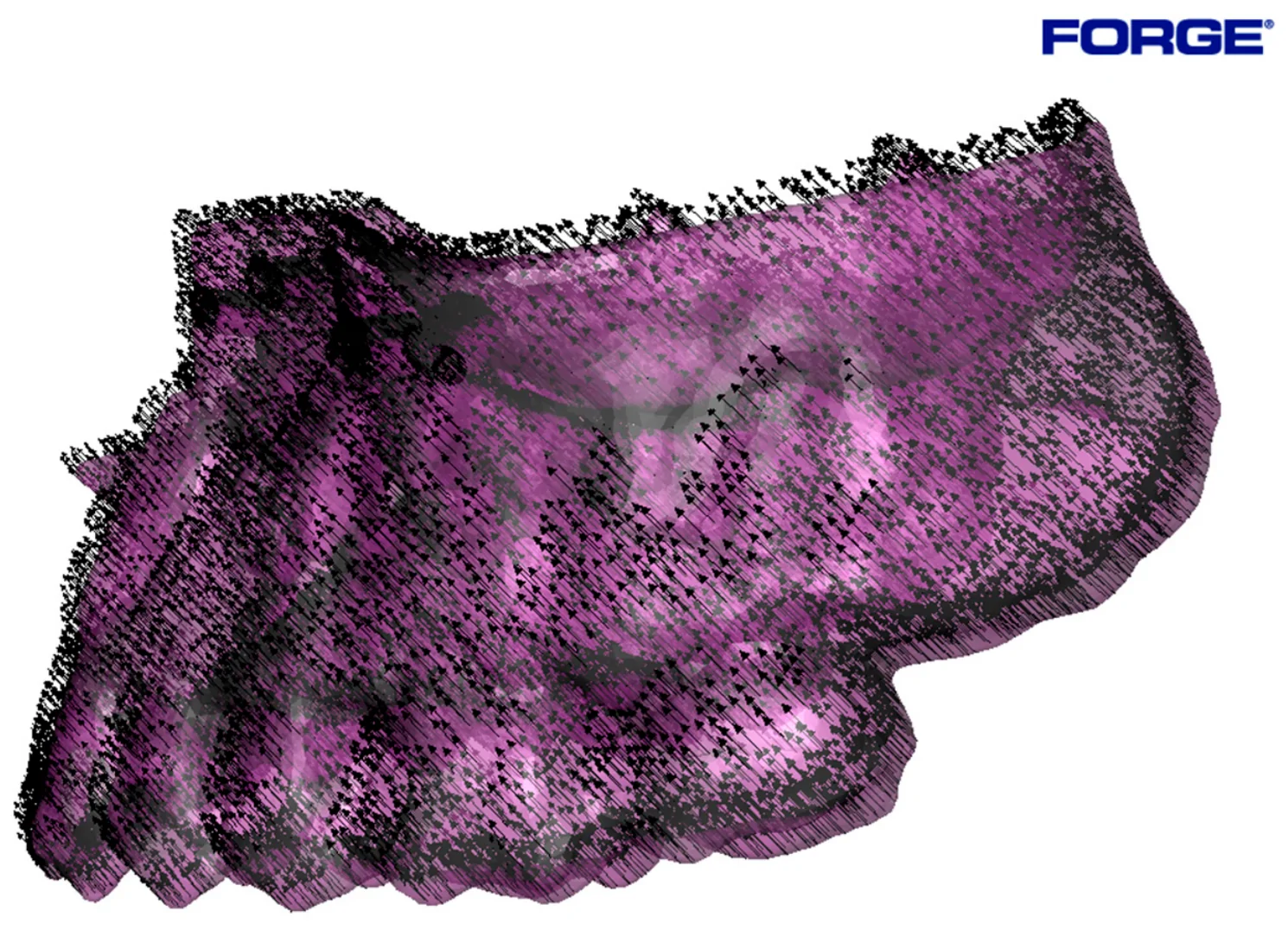
Displacement vectors analysis of the maxilla under postoperative loading. Displacement vectors illustrate a forward rotation tendency of the maxillary segment.

Several hypotheses may explain these findings. First, the multiarm architecture of the PSI likely improves stress dispersion. Plate geometry has been shown to strongly influence mechanical strength [32]. In our models, displacement was consistently higher in the posterior maxilla, in line with Li et al. [26] and Wu et al. [39], who reported greater movement in this region due to its distance from fixation points. The pterygomaxillary pillar remains a mechanical “blind spot” even with PSIs. Li et al. [26] and Huang et al. [7] reported that oblique masticatory loads tend to induce forward rotation of the maxilla, which was confirmed in this study, as illustrated by the displacement vectors of the maxilla (Figure A2).

**Figure 9:**
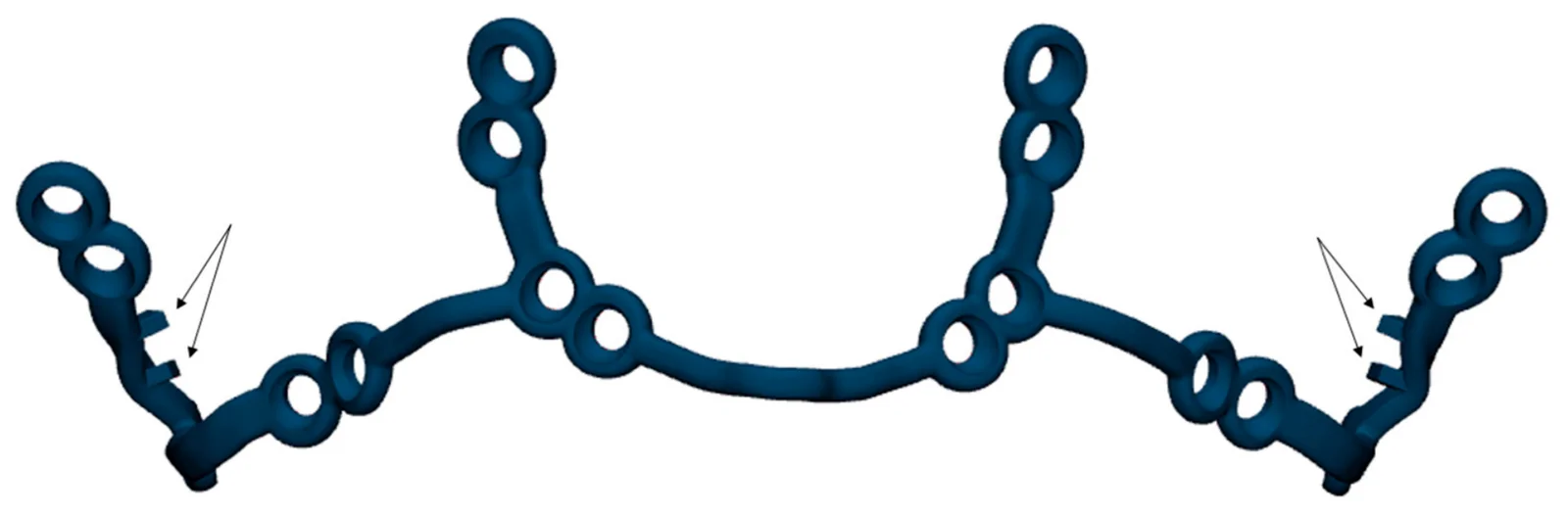
Modified multiarm PSI for patients requiring posterior impaction and clockwise rotation. Note the small devices added laterally on both sides (arrows).

Forward rotation may lead to counterclockwise relapse or loss of planned impaction. Although PSIs were initially expected to better control this phenomenon, Patient 2, whose surgical plan included posterior impaction together with greater maxillary advancement, exhibited greater bone stress and slightly higher displacement with PSI fixation. Several factors may have contributed to this model-specific response, including the magnitude and direction of maxillary repositioning, individual anatomy and bone geometry, as well as the mechanical behavior of the one-piece PSI. The greater rigidity and continuous architecture of the PSI may redistribute rather than simply reduce mechanical stress, transferring part of the load from the fixation device to the surrounding bone, whereas conventional miniplates deform locally, beginning with the posterior plates and subsequently the anterior ones. This trend toward loss of posterior impaction has been observed clinically in patients undergoing large advancements with clockwise rotation and impaction at our Center. To address this, small lateral devices have recently been added to the multiarm PSI to better resist posterior settling and enhance impaction stability (Figure 9). Importantly, the increase in maximum maxillary displacement observed with PSI fixation in Patient 2 was only 2.7 µm (16.9 vs. 19.6 µm). This difference is a numerical output of the model, and its clinical relevance cannot be established from the present study; it should therefore not be interpreted as evidence of clinically meaningful instability. A dual-component PSI design could also be explored to provide greater flexibility in combined movements. These observations highlight the importance of adapting PSI geometry to the planned surgical movements.

Second, although the elastic moduli of the two plates are almost similar, they are manufactured from different titanium grades and via different processes. Selective laser melting (PSI) and conventional machining (conventional plates) produce distinct microstructures, which may affect mechanical response under load. These parameters could not be incorporated into the present numerical model but remain mechanically relevant. Additionally, PSIs typically require two more screws than conventional plates. Prior studies indicate that the number and location of screws may be more influential than plate thickness in large advancements [32], and screw placement in regions of optimal bone thickness improves anchorage stability [40]. Screw insertion technique may also influence primary fixation stability in clinical conditions. In the present FE models, however, plates and screws were represented as a single continuous component, without explicit modelling of drilling, threading or locking mechanisms. Consequently, potential differences related to self-drilling versus predrilled screw insertion could not be assessed. Clinically, the screws used in the present cases were non-self-drilling. The anterior bridge of the PSI was modeled and in contact with bone surface, consistent with its clinical design. While useful in segmental osteotomies to maintain transverse dimension, its mechanical role in one-piece Le Fort I osteotomies may be limited. Further work is required to determine how variations in PSI architecture influence strain patterns, particularly in minimally invasive or segmented techniques. Moreover, the present model does not account for residual stress from plate bending, which likely underestimates the stress borne by conventional plates.

To our knowledge, this is the first study to evaluate the biomechanical behavior of the multiarm patient-specific maxillary plate, which has been used in Europe for nearly a decade. Earlier studies focused on long-term stability [18,19] and accuracy [9,10,17,20,21] and demonstrated that PSIs reproduce planned maxillary repositioning with high precision and stability comparable to that of conventional plating. The multiarm configuration may be particularly beneficial in segmental Le Fort I osteotomies, where transverse relapse is a concern due to bone segment recontraction. However, stability remains strongly associated with the extent of maxillary advancement, with large movements carrying increased relapse risk [3,7,14,15,16]. Whether PSIs could mitigate this risk warrants further investigation. Pozzer et al. [41] suggested potential advantages in vitro, although their loading conditions differed from physiological masticatory function. Beyond mechanical performance, PSIs offer practical advantages, including shorter operative time due to the absence of intraoperative plate bending and potentially fewer complications such as blood loss or infection. Patient-specific designs may also combine osteosynthesis with aesthetic contour optimization within a single device, for example, to enhance mandibular angle definition or correct contour irregularities [42,43]. However, these benefits come at the cost of increased preoperative planning and the need for osteotomy guides, as well as higher financial cost. However, these benefits come at the cost of increased preoperative planning and the need for osteotomy guides, as well as higher financial cost.

Like conventional plates, PSIs are made of titanium, an excellent but stiff material whose Young’s modulus remains ten times higher than that of bone. Emerging titanium alloys such as grade 23 (Ti6Al4V-ELI) or Near-Beta Ti (Ti-14Zr-13Nb Near-ß) may offer more bone-like stiffness, but remain investigational [44]. The use of metals for osteosynthesis has several consequences. First, although the titanium used for osteosynthesis is compatible with MRI, it is responsible for artifacts during MRI and CT scans. Second, the need to remove material remains subject to ongoing debate. Higher concentrations of titanium have indeed been detected in tissues attached to implant surfaces and in regional lymph nodes [45,46]. Some authors have proposed the use of resorbable plates made of poly L-co-DL lactic acid [39,47,48], but these plates are known to cause inflammatory reactions during degradation. Ueki et al. also reported a tendency toward maxillary spontaneous impaction with the use of resorbable plates [49]. Hingsammer et al. [50] studied the feasibility of zirconium dioxide ceramic miniplates. However, they reported an increased risk of fracture compared with titanium implants due to low fracture toughness, as well as stress shielding due to a very high elastic modulus (210 GPa).

A key strength of this study lies in the use of real surgical plans, as subject-specific FE models, to compare two fixation methods. Most FEM studies evaluate a single patient model with standardized displacements, whereas our models reflect clinically optimized movements defined by cephalometrics, occlusion, and soft-tissue objectives. This approach aligns with surgical reality: alternative movements would not have been clinically relevant for the patients studied. Interestingly, the contrasting results observed between patient 1 and patient 2 underscore the influence of interindividual anatomical variability -including maxillary geometry, bone quality and movement magnitude- on fixation biomechanics. This supports the value of subject-specific FEA as a tool for biomechanical prediction and for selecting or adapting fixation strategies within a personalized plan.

Choi et al. reported that patients with malocclusion have a lower muscle bite force than nondysmorphic patients do [37]. They also reported that surgery does not completely normalize the bite force but can harmonize the stress distribution on the jaw and facial skeleton. In this study, loading conditions were selected to reflect the early postoperative period, when fixation system absorbs the entire mechanical load. Although more personalized approaches would include patient-specific postoperative forces, such measurements are difficult to obtain immediately after surgery. Von Mises stress was chosen as the primary metric because it reflects the risk of plastic deformation in ductile materials such as titanium. While principal stresses provide complementary information, von Mises stress remains the standard for evaluating fixation performance under multiaxial loading. Alongside Janovic et al. [51], the magnitude of von Mises stress in our model was significantly lower than that in previous FE models consisting only of cortical bone. This finding supports their conclusion that both cortical and trabecular bone should be modeled when stress is analyzed quantitatively.

This study has several limitations. First, teeth were voluntarily not modeled as separate structures as they lie relatively far from the osteotomy site; however, they could absorb part of the applied load through the periodontal ligament. Bone was modeled as linear, elastic and homogeneous, despite its anisotropic nature. These simplifications, commonly used in orthognathic FE studies [7,13,26,27], were necessary for numerical convergence.

Second, the model considered only one type of occlusal loading; additional axes or dynamic mandibular movements (e.g., via Modjaw^®^ [52]) could further refine predictions but are difficult to collect in early postoperative conditions. Notably, the model does not account for certain physiological or nonphysiological forces: facial muscle forces are typically excluded from FE studies because of their minimal role, which is further diminished by surgical dissection and postoperative swelling. Elastics forces, ranging from 1 to 1.8 N (3.5 to 6.5 oz), are also negligible in a numerical model. Finally, although preliminary convergence testing included comparison with a friction-contact formulation, sliding contact was retained for the final simulations. Because several previous FE studies used friction contact with a coefficient of 0.5, absolute displacement values should be compared cautiously across studies using different contact formulations. Importantly, both fixation strategies were evaluated under identical contact conditions within each subject-specific model, preserving the internal comparison between PSI and conventional fixation.

Third, the maximum maxillary advancement in this study was approximately 5 mm; larger movements, although less frequent, may produce different biomechanical responses and should be investigated in future work. Importantly, the two subject-specific models differed in several respects, including craniofacial anatomy, malocclusion type, magnitude of maxillary advancement, and the presence of posterior impaction in Patient 2. Consequently, the contribution of posterior impaction cannot be isolated from these other patient-specific factors. The less favorable bone stress distribution observed in Patient 2 should therefore be considered a model-specific finding rather than evidence of a causal effect of posterior impaction. Additionally, Patient n°1 received two bone grafts at the anterior part of the maxilla. We have chosen not to consider them to increase the comparability of the models. However, Fahradyan et al. reported that bone grafting could help reduce the risk of relapse because it promotes faster bone healing [14]. The present model represents the mechanical environment immediately following surgery, prior to bone healing. During this phase, the fixation system bears the entire load, making it the critical window for evaluating fixation reliability.

Fourth, the number of subjects in this study was limited to two patients. Although this reflects standard FEM methodology, broader generalization will require additional anatomical scenarios. Finally, no statistical tests were performed since the results are based on deterministic numerical simulations and not on probabilistic or empirical sampling. In finite element modeling, each scenario is defined by precise boundary conditions and material properties, and the outputs are uniquely determined.

Future research should extend this subject-specific framework to a broader range of anatomical and surgical scenarios, including larger advancements, impaction, and segmental Le Fort I osteotomies, as well as alternative PSI architectures. More physiologically detailed models could incorporate patient-specific functional loading and dynamic mandibular motion. Ultimately, prospective correlation between FE-predicted biomechanical patterns and postoperative clinical stability would be valuable to determine whether preoperative biomechanical simulation can contribute to patient-specific optimization of fixation design.

## 5. Conclusions

In this subject-specific finite element study based on real surgical plans, patient-specific multiarm maxillary plates reduced implant stress while maintaining maxillary stability comparable to conventional miniplates under the simulated static loading conditions. However, their effect on surrounding bone stress differed between the two subject-specific models. In the model including greater maxillary advancement and posterior impaction, PSI fixation was associated with higher bone stress and a small numerical increase in displacement, whose clinical relevance remains uncertain. Because the models also differed in anatomy and surgical characteristics, these findings cannot establish an independent effect of posterior impaction. Further investigations across a larger range of subject-specific models are required to determine how patient anatomy and surgical movements interact with PSI geometry and fixation biomechanics. These results support the potential value of integrating biomechanical simulations alongside anatomical personalization in orthognathic surgical planning.

## Figures and Tables

**Table 1:** Patients’ characteristics. ANS: anterior nasal spine, the extreme anterior point of the maxilla, reflecting relative maxillary advancement. CWR: clockwise rotation of the occlusal plane.

Characteristics	Patient n°1	Patient n°2
Age (years)	26	30
Sex	Male	Female
Malocclusion type	II	III
Type of surgery	Bimaxillary orthognathic surgery with CWR	Bimaxillary orthognathic surgery with CWR and posterior impaction
Relative ANS advancement (mm)	1.65	4.94

**Table 2:** Plates’ characteristics.

Type of Plate	Grade of Titanium	Alloy Type	Mechanical Resistance/Tensile Strength Rm (MPa)	Elastic Limit/Yield Strength Re (MPa)	Elongation at Break A %	Density g/cm^3^	Beta Transus	Melting Temperature °C	Thickness (mm)	Total Number of Screws
Conventional miniplates	2	Commercially pure titanium UNS R50400 (T40)	485	345	28	4.54	948°	1660	2	16
PSI	5	Ti6Al4V	897	795	14	4.42	999°	1660	2	18

**Table 3:** Number of elements and nodes for each FEM.

	Patient n°1		Patient n°2	
	Number of Elements	Number of Nodes	Number of Elements	Number of Nodes
Models with conventional miniplates	5.42 × 10^6^	1.10 × 10^6^	3.43 × 10^6^	7.24 × 10^5^
Models with PSI	5.53 × 10^6^	1.12 × 10^6^	3.49 × 10^6^	7.44 × 10^5^

**Table 4:** Mechanical parameters of the materials used for the FE model.

Materials	Elastic Modulus (GPa)	Poisson’s Ratio	Density
Cortical bone	14.0	0.3	1.3
Cancellous bone	1.37	0.3	1.3
Conventional miniplates	114.0	0.34	4.54
Patient-specific plates	116.1	0.32	4.42

**Table 5:** Maximum displacements, in micrometres (µm), of the maxilla, the midface and the complex plate + screws of each 3D model.

	Patient n°1 Maximum Displacements (µm)			Patient n°2 Maximum Displacements (µm)		
	Maxilla	Midface	Plate	Maxilla	Midface	Plate
Fixation with conventional miniplates	12.8	6.72	10.5	16.9	11.2	13.5
Fixation with PSI (difference)	12.9 (+0.1)	7.03 (+0.31)	9.99 (−0.51)	19.6 (+2.7)	12.3 (+1.1)	15.2 (+1.7)

**Table 6:** Maximum von Mises stress, in MPa, on the plates and screws of each 3D model.

	Patient n°1	Patient n°2
Fixation with conventional miniplates	107.4	140.7
Fixation with PSI	61.3	67

**Table 7:** Maximum von Mises stress (MPa) on bone segments.

	Patient n°1 Maximum Stress (MPa)		Patient n°2 Maximum Stress (MPa)	
	Maxilla	Midface	Maxilla	Midface
Fixation with conventional miniplates	19.7	58.1	15	27.2
Fixation with PSI (difference)	9 (−10.7)	9.7 (−48.4)	25.2 (+10.2)	40 (+12.8)

## Data Availability

The data supporting the findings of the present study are available from the corresponding author upon request.

## Abbreviations

The following abbreviations are used in this manuscript:

3D	Three-dimensional
ANS	Anterior nasal spine
CAD-CAM	Computer-aided design/computer-aided manufacturing
CBCT	Cone-beam computed tomography
CWR	Clockwise rotation
DICOM	Digital Imaging and Communications in Medicine
FE	Finite element
FEA	Finite element analysis
FEM	Finite element model/modelling
PSI	Patient-specific implant
SNA	Sella-nasion-A point angle
STL	Standard tessellation language

## Appendix A

**Table A1:** Planned movements. A: Point A or subspinale point A, the deepest point on the curvature of the maxillary alveolar process; ANS: anterior nasal spine, the extreme anterior point of the maxilla; SNA: the angle between the sella (S), the nasion (N) and the A point (A), which determines the anteroposterior relationship of the maxilla relative to the cranial base.

	Patient n°1			Patient n°2		
Point	Delta X (R)ight/(L)eft	Delta Y (Ant)erior/(Post)erior	Delta Z Up/Down	Delta X (R)ight/(L)eft	Delta Y (Ant)erior/(Post)erior	Delta Z Up/Down
A	1.05 R	1.32 Ant	3.64 Down	0.00	4.47 Ant	0.30 Up
ANS	1.07 R	1.65 Ant	3.69 Down	0.00	4.94 Ant	0.00
Maxilla Incisor Midline	1.03 R	0.09 Ant	3.86 Down	0.00	3.00 Ant	0.00
Maxilla Canine L	0.97 R	0.22 Ant	3.29 Down	0.00	2.97 Ant	0.51 Up
Maxilla Canine R	0.97 R	0.01 Post	3.32 Down	0.00	3.00 Ant	0.61 Up
Maxilla 1st Molar L	0.82 R	0.38 Ant	1.87 Down	0.00	3.06 Ant	1.82 Up
Maxilla 1st Molar R	0.82 R	0.02 Post	2.02 Down	0.00	3.05 Ant	1.85 Up
Maxilla 2nd Molar L	0.75 R	0.51 Ant	1.18 Down	0.00	3.16 Ant	2.53 Up
Maxilla 2nd Molar R	0.74 R	0 Post	1.27 Down	0.00	3.14 Ant	2.58 Up
	Pre-Op 3D	Planned 3D	Delta 3D	Pre-Op 3D	Planned 3D	Delta 3D
SNA (°)	84.8	85.7	0.9	79.9	84.4	4.5
